# Autonomous driving using imitation learning with look ahead point for semi structured environments

**DOI:** 10.1038/s41598-022-23546-6

**Published:** 2022-12-09

**Authors:** Joonwoo Ahn, Minsoo Kim, Jaeheung Park

**Affiliations:** 1 Dynamic Robotic Systems (DYROS) Lab., Graduate School of Convergence Science and Technology, Seoul National University, 1, Gwanak-ro, Seoul, 08826 Republic of Korea; 2 ASRI, RICS, Seoul National University, Seoul, Republic of Korea; 3 Advanced Institutes of Convergence Technology, Suwon, 443-270 Republic of Korea

**Keywords:** Engineering, Electrical and electronic engineering

## Abstract

Semi-structured environments are difficult for autonomous driving because there are numerous unknown obstacles in drivable area without lanes, and its width and curvature considerably change. In such environments, searching for a path on a real-time is difficult, and localization data are inaccurate, reducing path tracking accuracy. Instead, alternative methods that reactively avoid obstacles in real-time using candidate paths or an artificial potential field have been studied. However, these require heuristics to identify specific parameters for handling various environments and are vulnerable to inaccurate input data. To address these limitations, this study proposes a method in which a vehicle drives toward drivable area using vision and deep learning. The proposed imitation learning method learns the look-ahead point where the vehicle should reach on a vision-based occupancy grid map to obtain a safe policy with a clear state action pattern relationship. Furthermore, using this point, the data aggregation (DAgger) algorithm with weighted loss function is proposed, which imitates expert behavior more accurately, especially in unsafe or near-collision situations. Experimental results in actual semi-structured environments demonstrated the limitations of general model-based methods and the effectiveness of the proposed imitation learning method. Moreover, simulation experiments showed that DAgger with the weight obtains a safer policy than existing DAgger algorithms.

## Introduction

Autonomous driving technology for semi-structured environments such as parking lots and alleyways is important for fully autonomous driving. Moreover, it is more difficult than driving in structured environments. In a structured environment, autonomous driving involves a global plan using a road network, and a vehicle keeps within a lane via lateral control and maintains a safe distance from vehicles in front while following a target speed through longitudinal control. Furthermore, in an semi-structured environment, the curvature can rapidly change, such as at right-angled corners, and the drivable area can be narrowed because of double-parking or illegal parking. Other obstacles include vehicles, humans, curbs, and bollards, which vary in shape, size, and location. Typically, such obstacles are unknown in advance. Navigating such a situation is difficult even in a static environment, and existing motion-planning algorithms are unable to handle such settings.

**Figure 1:**
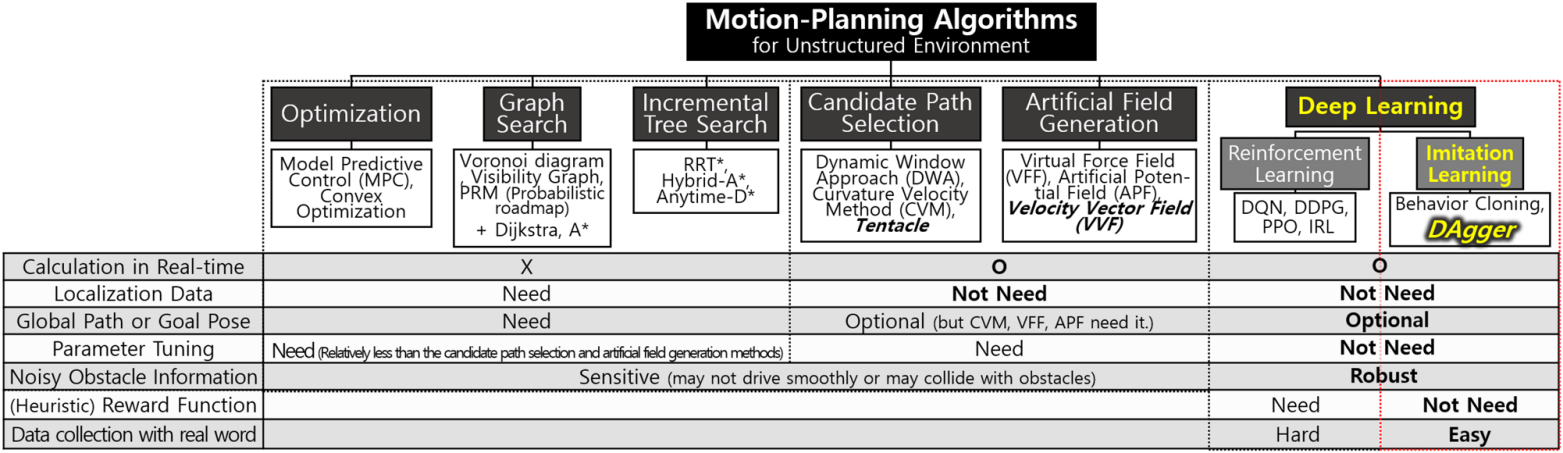
Motion-planning algorithms and comparison between the characteristics.

A representative approach for driving in semi-structured environments is to generate a global map on a global path to reach the destination. The vehicle tracks the path using localization data (i.e., the position and heading of the vehicle relative to the path). While tracking the global path, the vehicle checks for obstacles in its path. Object detection algorithms detect the position and shape of obstacles using camera or LiDAR sensors with pattern recognition or deep learning. If obstacles are detected near the global path, motion-planning is used to search a local path or waypoint that can reach the global path without collision. These motion-planning algorithms developed for robotics have been used in autonomous vehicles^1^. These can be categorized according to the method and calculation time. An overview of motion-planning algorithms is shown in Fig. 1.

The path planning method using optimization theory, such as model predictive control (MPC)^2^ and convex optimization^3^, uses a vehicle’s kinematic or dynamic model to predict its future trajectory. This method provides an optimal solution that satisfies the objective function and constraints. In driving situations, the objective function can be modeled as avoiding obstacles while reaching the global path and maintaining the target speed. Constraints can be the control capabilities and maintaining a safe distance from obstacles. The graph-search path planning method builds a graph in the local area and then searches for a path. The Voronoi diagram^4^, Visibility graph^5^, and Probabilistic roadmap (PRM)^6^ algorithms can be used to build the graph. These algorithms discretize the configuration space into obstacles and free space, which are represented in the form of a graph. The graph is used to search for the minimum path length using the Dijkstra or A* graph search algorithm. The searched path is interpolated via spline algorithms to satisfy vehicle constraints and obtain a smooth path. An incremental search path planning method uses tree exploration algorithms, which iteratively expand a tree into free space at the end of the tree reaches a goal. Rapidly exploring random trees$$^*$$ (RRT$$^*$$) algorithm^7^ extends the tree with samples randomly selected in the configuration space. The hybrid-A$$^*$$^8^ and anytime-D$$^*$$^9^ algorithms expand the tree in grid units. The shortest path is searched for to reach the goal pose while satisfying the non-holonomic constraints of the vehicle. The non-holonomic constraint refers to a motion that cannot move directly sideways, so a vehicle must drive forward or backward to rotate. However, these methods have three problems^10^. First, if the local area is large or complex, a long computational time is required to generate the path, and the solution may not be found within a control loop (i.e., not real-time). Second, selecting a goal pose in the global path to search for the local path is heuristic. Third, when an algorithm is implemented, accurately recognizing whether an obstacle is close to the global path and tracking the path without collision is difficult because of inaccurate localization data. In semi-structured environments, various types of obstacles are complexly placed in the drivable area. Thus, obtaining accurate localization data at every point in semi-structured environments is difficult.

Rather than searching and tracking a path, alternative methods can be used that allow the vehicle to drive toward the global path while reactively avoiding obstacles. The candidate path selection and artificial field methods find a solution close to a vehicle that can be calculated in real-time. They select a candidate path or waypoint and calculate the control commands. The candidate path selection method generates candidate paths and selects one path that satisfies multiple objectives. These paths are smooth and are designed to account for the non-holonomic constraints of the vehicle. To select one path, the objective function is modeled to reach the global path, avoid obstacles, and keep the ride comfortable. Three algorithms have been used to achieve these: the dynamic window approach (DWA)^11^, the curvature velocity method (CVM)^12^, and tentacle^13^ algorithms. The DWA algorithm designs a window according to the current state of the vehicle, and candidate paths are generated within the window. The CVM algorithm is similar to DWA, and it additionally considers vehicle accelerations. The tentacle algorithm mimics the antennas of a beetle as candidate paths to drive on narrow and variable-curvature roads more smoothly than DWA and CVM. The artificial field method uses a repulsive field against obstacles and an attractive field toward the global path. These fields are combined with different weights, and a vehicle is guided by the combined field’s vector. There are three algorithms available that differ in how they model the fields: namely, the virtual force field (VFF)^14^, the artificial potential field (APF)^15^, and the velocity vector field (VVF)^16^ algorithms. The VFF algorithm calculates the repulsive force as a vector from the obstacle to the vehicle and the attractive force as a vector from the vehicle to the target point. The APF algorithm creates a repulsive field with high potential energy for obstacles and an attractive field with high energy at the vehicle point and low energy at the goal point. The *VVF* algorithm considers the desired velocity and velocity of obstacles, in addition to the fields of the APF algorithm. However, the candidate path selection and artificial field methods have several limitations that make them difficult to be used in semi-structured environments. First, the parameters in the objective function or field model may differ to cope with the various complex situations of semi-structured environments. It is difficult to identify specific parameters that can handle all of these situations. Second, inaccurate localization data make it difficult in practice to know where exactly the global path is located in a local area. Third, if the local obstacle information is difficult to recognize accurately especially at road boundaries or shadowed areas (i.e., noisy state), the vehicle may not drive smoothly^17^. Moreover, a vehicle may drive out of drivable area or toward an obstacle.

To address these limitations, this study proposes an imitation learning-based method for selecting the look-ahead point to drive toward the drivable area while avoiding obstacles in real-time without the use of global information such as the global map and localization data. The proposed method segments vision data into the drivable area and non-drivable area using deep learning, and this is represented as an occupancy grid map; it does not recognize whether obstacles exist close to the global path and can ignore irrelevant information for driving, improving the generality of driving policy in untrained environments. Imitation learning obtains a safe driving policy by collecting expert driving data for various complicated situations that occur in semi-structured environments such as large changes in the curvature and width of the drivable area. Therefore, it is not necessary to manually model the policy and tune parameters heuristically to handle such situations. The data include cases where the occupancy grid map is incorrectly recognized correctly or is noisy because of shadows, ensuring that the driving policy is robust in these situations. Even with a human-in-the-loop design, an imitation learning algorithm (DAgger) can obtain the policy faster than a reinforcement learning algorithm. This is because reinforcement learning requires trial-and-error and heuristic reward function modeling^18^, but with imitation learning, the algorithm can directly use the collected expert data. Additionally, reinforcement learning can typically only be applied through a simulator (which enables trial-and-error learning).

The proposed imitation learning method trains the driving policy to select the look-ahead point on the occupancy grid map. The look-ahead point is a target waypoint for a vehicle to reach, which is calculated from the pure pursuit algorithm^19^ that is commonly used in autonomous driving. There are several advantages to using the look-ahead point. First, selecting the look-ahead point that can avoid obstacles while driving fast on the occupancy grid map has a clearer pattern relationship than a front-view image and steering-velocity relationship which is common in imitation learning. The driving policy can properly train driving patterns and is safer. Second, the trained driving policy and expert behavior can be shared, allowing the data aggregation (*DAgger*) algorithm^20^ to be applied to the autonomous vehicle. *DAgger* enhances the imitation learning performance, which retrains the policy by collecting additional data. Furthermore, a new *DAgger* training method, *DAgger* with the weighted loss function (*WeightDAgger* algorithm), is proposed to accurately imitate expert’s look-ahead point, particularly in unsafe or near-collision situations than existing *DAgger* algorithms. The weight values were calculated using the action discrepancy between the trained policy and expert look-ahead points obtained during *DAgger*. These were paired with the additional data sampled by *DAgger* and the entire training dataset with a high state similarity. By using the weight, the policy is trained with a high learning rate for high-discrepancy data where the trained policy cannot cope well. The *WeightDAgger* algorithm is simple, but it is an meaningful finding to calculate the action discrepancy represented to a single scalar value by performing imitation learning with the look-ahead point.

Our contributions are summarized as follows:

- A method is proposed to drive with only vision data in semi-structured environments using imitation learning, which does not use high-cost HD-map and inaccurate localization data in a complex environment.
- Compared to other *DAgger* algorithms, *DAgger* trained with the look-ahead point have two advantages; applicable to autonomous driving without an additional joystick device; Even when the vehicle is controlled by the trained policy action (even in human-in-loop design), the expert can select the optimal action well.
- A new *DAgger* training method, *DAgger* with the weighted loss function (*WeightDAgger*), is proposed to accurately imitate the look-ahead point in unsafe or near-collision situations and to achieve the desired policy with fewer human effort and fewer *DAgger* iterations.
- Real-world experiments show the limitations of the model-based motion-planning algorithms and the effectiveness of the proposed method, which is robust to sensor noise and does not require tuning model parameters to handle various and complex environments.

## Methods

This section presents methods for obtaining the occupancy grid map from vision data and a safe driving policy in an semi-structured environment through imitation learning. The input for imitation learning is the occupancy grid map, and the output is the look-ahead point used to control the vehicle. This study assumes that the road has only static obstacles and no intersections. In order to navigate intersections based on the proposed method, a branch road detection method^21^ can be referred.

### Vision-based occupancy grid map

**Figure 2:**
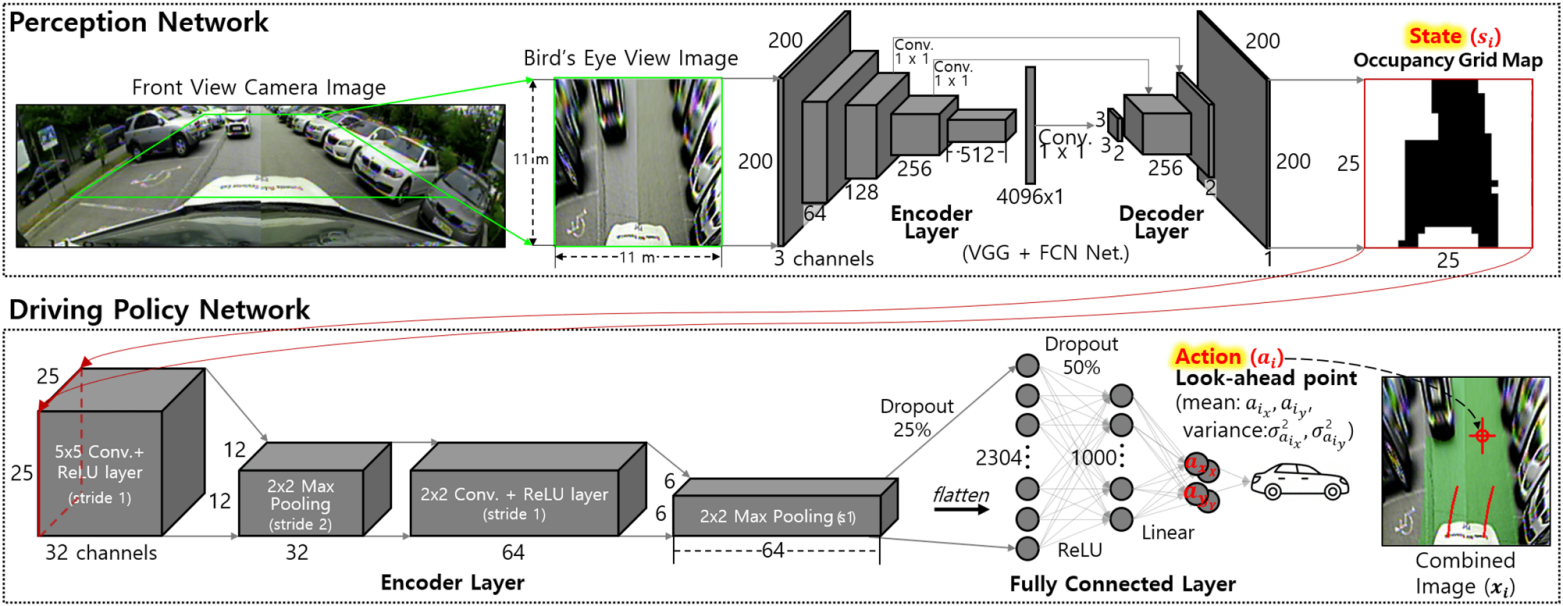
System architecture and deep neural network of the proposed methods.

The occupancy grid map is a two-dimensional map that divides an area into a grid (see Fig. 2). Each grid in the map contains information on whether it is occupied (non-drivable) or unoccupied (drivable). It serves as the input for motion-planning algorithms, which have two advantages: First, the segmented image can ignore irrelevant information for driving, such as differences in the types of obstacles and sidewalks in the drivable area. Therefore, driving policies can achieve similar performance in untrained environments, which can enhance the generality of driving performance. Second, close and far distance information can be distinguished because the occupancy grid map is a 2D map (i.e., bird’s-eye-view). Thus, the vehicle can avoid nearby obstacles preferentially or consider distant obstacles in advance.

The front view camera image is corrected using intrinsic and extrinsic parameters and is transformed into a bird’s-eye-view image through the warp perspective function of the OpenCV library. The transformed image is segmented into drivable and non-drivable areas with a deep neural network through semantic segmentation. The drivable area is defined as the road, crosswalk, and road markings. The non-drivable area includes the road boundary lines, sidewalks, parking spaces with lines, pedestrians, and obstacles. The 200 $\times$ 200 segmented image is divided into 8$^2$ pixels per grid to obtain a 25 $\times$ 25 grid map. If all pixel values within a grid are drivable, the grid is considered non-occupied.

The perception network is similar to the segmentation task of MultiNet^22^, which is based on the U-Net structure. The encoder is the same as that of the VGG network^23^ except for the last layer, comprising five pairs of convolutional and max-pooling layers, which is used to extract several abstract features from the input image. Then, one 1 $\times$ 1 fully-connected layer is connected at the end. The encoder’s output is passed through a 3 $\times$ 3 convolutional layer before being up-sampled with three transposed convolution layers. At this point, each convolutional layer of the encoder is combined with the decoder through the skip connections to extract high-resolution features from the encoded low-resolution features.

### Imitation learning method for autonomous driving in semi-structured environment

Imitation learning involves mimicking the behavior of an expert in a certain state. While an expert is driving, state action pairs of data are collected. The driving policy $\pi _{net}$ (i.e., deep neural network) is trained with the data through a process known as *behavior cloning*, which is a single training step in imitation learning. To address the limitations of *behavior cloning*, *DAgger*^20^ is used to collect additional data by executing the trained *behavior cloning* policy and retraining $\pi _{net}$. This process is repeated until the best policy is obtained. Furthermore, the weighted loss function is used in the *DAgger* training process to increase the accuracy of data for unsafe or near-collision situations.

#### Proposed imitation learning composition

The dataset comprises state and action pairs $D=\{(s_t, a_t)\}_t$, where *t* is an index of the data. The state $s_t$ is the occupancy grid map (25 $\times$ 25 grid $\in$ {***0*** (black): drivable(unoccupied), ***1*** (white): non-drivable(occupied)}), which is used for the input of the driving policy $\pi _{net}$. The action $a_t$ is a command of an expert and the output of $\pi _{net}$. In this study, the look-ahead point was used as the action $a_{t}$ $\in$ {$a_{t_x}$, $a_{t_y}$}, which is the target waypoint for a vehicle to reach. Most autonomous driving studies based on imitation learning use the steering-accel/brake as the action, but the look-ahead point is more useful in executing the proposed *DAgger* algorithm. This reason is explained in detail in Section 10. The output of the policy for a state is expressed as follows: $a_{net, t} = \pi _{net}(s_t)$, where $a_{net, t}$ $\in$ {$\bar{a}_{net, t_x}$, $\bar{a}_{net, t_y}$, $\sigma ^2_{a_{net, t_x}}$, $\sigma ^2_{a_{net, t_y}}$} are the mean and variance of the look-ahead point. The variance of the look-ahead point is calculated using Gaussian process (GP) to quantify the uncertainty or confidence of $\pi _{net}$^24^.

**Figure 3:**
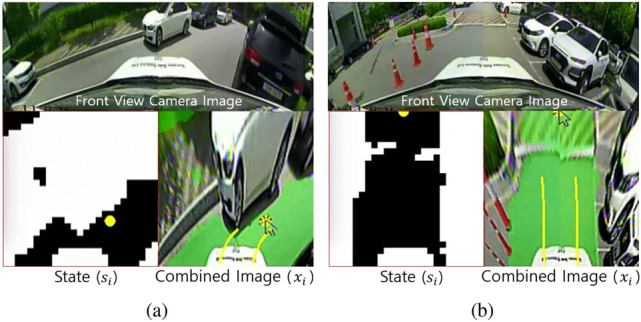
Dataset collection process of behavior cloning. The yellow look-ahead point is the action $a_{exp, t}$ selected by an expert. The expert selects $a_{exp, t}$ in the combined image $x_t$. The yellow lines are the future trajectory that the vehicle will drive towards $a_{exp, t}$ along during a certain time.

**Figure 4:**
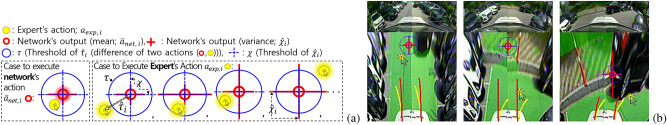
Illustration of the data-sampling function of *WeightDAgger*: (**a**) Whether executing the network’s action ($\bar{a}_{net, t}$) or the expert’s action ($a_{exp, t}$). (**b**) Unsafe or near-collision situations and collecting the additional dataset. In this example, *DAgger* is in iteration *i* = 1, and the network $\pi _{net, i=1}$ has the *behavior cloning* policy, $\pi _{BC}$. The yellow point is the newly labeled action $a_{exp, t}$ of the expert while $\pi _{BC}$ is being executed. The red point in the combined image $x_t$ is the mean of the output by $\pi _{net, i}$: the network’s action $\bar{a}_{net, t}$. The blue circle is the threshold $\tau$ of $\hat{\tau }_t$ which is the difference between the actions $\bar{a}_{net, t}$ and $a_{exp, t}$. The red lines centered at $\bar{a}_{net, t}$ represent the variance of the output of $\pi _{net, i}$: $\hat{\chi }_t$. The blue dashed lines centered at $\bar{a}_{net, t}$ represent the threshold of $\hat{\chi }_t$ which is the variance of the output of the network $\pi _{net, i}$: $\chi$.

To collect training data, the expert selects the look-ahead point $a_{exp, t}$ $\in$ {$a_{exp, t_x}$, $a_{exp, t_y}$}, and the vehicle is controlled in real-time to reach the selected look-ahead point. The pure pursuit algorithm^19^ is used to calculate the steering angle command. The velocity command is proportional to the distance between this point and the vehicle. The dataset $D = \{(s_t, a_{exp, t})\}_t$ is stored for every period *t* as the vehicle is moving, and numerous data can be easily collected. This process is repeated continuously until the driving is completed. As shown in Fig. 3, the expert selects the look-ahead point $a_{exp, t}$ using a mouse pointer in the combined image $x_t$ instead of the occupancy grid map, $s_t$: $a_{exp, t} = \pi _{exp}(x_t)$, where $\pi _{exp}$ indicates the behavior of the expert. The combined image $x_t$ is an image of transparently combining the information about the drivable area to the *RGB* image: $x_t$ $\in$ {*RGB with green*: drivable, *RGB only*: non-drivable}, which is because, if $s_t$ is inaccurate (i.e., noisy), the expert may wrongly select the look-ahead point. This situation is shown in Figs. 3b and 4b.

The selection of the look-ahead point in the state (occupancy grid map $s_t$) takes into account the distribution of obstacles and the drivable area. With this, the following three criteria can be proposed to driving in semi-structured environments, which the expert can refer to and select the look-ahead point $a_t$: (i) The look-ahead point must be within the drivable area. (ii) The expert selects the look-ahead point where obstacle avoidance is possible by referring to future trajectories calculated on the basis of the kinematic bicycle model indicated in Figs. 3 and 4b. (iii) The look-ahead point is selected as far as possible while satisfying the first and second conditions so that the vehicle can move fast. Based on these criteria, it is not difficult for experts to label actions that allow the vehicle to avoid obstacles and drive toward the drivable area as fast as possible. For example, if an obstacle exists on the front and left side of a vehicle, the look-ahead point is selected to be on the right and near the front side of the vehicle in the drivable area (see Fig. 3a). At this point, a large steering angle and low-velocity command are calculated, and the vehicle can safely avoid obstacles. Conversely, if there are no obstacles, the look-ahead point is chosen as far as possible from the vehicle in the drivable area (see Fig. 3b). At this point, the vehicle can drive at high speed with a small steering angle difference.

#### Behavior cloning

The collected data can be used to train the policy $\pi _{net}$ in a process similar to that of supervised learning. $\pi _{net}$ is expressed as $\pi _{net}(s_t; \theta )$ parameterized by $\theta$ for the state $s_t$. The process of training $\pi _{net}(s_t; \theta )$ is the process of optimizing $\theta$ to minimize the loss function $\mathscr {L}_{Gau_t}$ for $s_t$. This is expressed as $\mathscr {L}_{Gau_t}(\pi _{net}(s_t; \theta ), a_{exp, t})$, and its detailed expression is given in (1). A large number *T* of datasets $D = \{(s_t, a_{exp, t})\}^N_{t = 1}$ is used to optimize $\theta$:

1 $$\begin{aligned} \underset{\theta }{min}\ \, \sum ^T_{t=1} \mathscr {L}_{Gau_t}(\pi (s_t;\theta ), a_{exp, t}), \end{aligned}$$

where $\mathscr {L}_{Gau_t}$ is the multivariate Gaussian log-likelihood loss function. Through $\mathscr {L}_{Gau_t}$, the policy infers the mean and variance of the look-ahead point^24^:

2 $$\begin{aligned} \mathscr {L}_{Gau_t} = \frac{1}{n} \sum _j \frac{1}{2} \frac{\mathscr {L}_{t_j}}{\sigma ^2_{t_j}} + \frac{1}{2} \log |\sigma ^2_{t_j} |, \end{aligned}$$

where *n* is the dimension of the look-ahead point and *j* is the index of *n*, so *j* belongs to *x* and *y*, and *n* becomes two. $\mathscr {L}_{t_j}$ in $\mathscr {L}_{Gau_t}$ is the non-weighted loss function used to infer the look-ahead point:

3 $$\begin{aligned} \mathscr {L}_{t_j} = {(a_{exp, t_j}-\bar{\pi }_{net, a}(s_t;\theta )_j)}^2, \end{aligned}$$

where $\bar{\pi }_{net, a}(s_t;\theta )_j$ is the mean of the policy output (look-ahead point) while training; $a_{exp, t_j}$ is label of the look-ahead point. $\sigma ^2_{t_j}$ is the variance of the policy output:

4 $$\begin{aligned} \sigma ^2_{t_j} = {(0.0 - \bar{\pi }_{net, \sigma }(s_t;\theta )_j)}^2, \end{aligned}$$

where $\bar{\pi }_{net, \sigma }(s_t;\theta )_j$ is the policy output while training; 0.0 is a labeled variance value that the network is trained to output a low variance. When this process is performed only once, the trained policy $\pi _{net}$ is denoted as $\pi _{BC}$. When a vehicle drives with $\pi _{BC}$ in an environment similar to the trained environment, $\pi _{BC}$ calculates a look-ahead point similar to that of the expert.

However, if $\pi _{BC}$ encounters states that are not similar to dataset *D* or are noisy, $\pi _{BC}$ may output unsafe or unsafe actions. As shown in Fig. 4b, a noisy state is when the boundary of the drivable area or shadow area is not accurately recognized. Furthermore, the location and type of obstacles differ when the dataset for $\pi _{BC}$ is collected and executed. Here, the vehicle cannot sufficiently avoid obstacles; this is known as the *data mismatch problem*, which occurs when the data for unsafe or near-collision situations are included in the training dataset *D* less often than situations of driving in a relatively large drivable area or with no misrecognition problems. Thus, the policy $\pi _{net}$ cannot reflect these situations in $\pi _{BC}$ well; this is known as the *data imbalance problem*. Moreover, when these problems occur in a driving situation, the error may magnify afterward because $\pi _{BC}$ has not learned recovery behavior; that is known as the *compounding error problem*.

#### DAgger algorithm

The *DAgger* algorithm can be used in imitation learning to address the problems of *behavior cloning*^20^. *DAgger* aggregates an additional dataset $D_i$ with the previously collected dataset *D* and trains the policy $\pi _{net}$ again. This process is repeated until the desired policy is obtained.
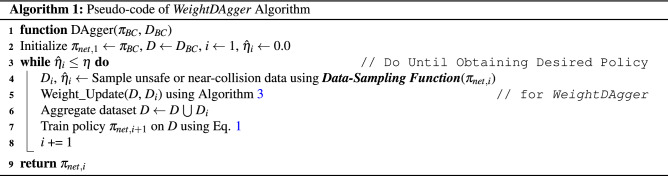


Algorithm 1 represents the basic structure of *DAgger*. First, *DAgger* initializes the policy $\pi _{net, i=1}$ and dataset *D* as those obtained from *behavior cloning*. The *DAgger* iteration *i* and $\hat{\eta }_i$ representing the performance of the trained policy $\pi _{net, i}$ are initialized. When the iteration is started (*i* = 1), the additional dataset $D_i$ is collected by the *data-sampling function* as described in the next subsection (line 4 in Algorithm 1), which samples the only data for unsafe or near-collision situations. Following the driving via the *data-sampling function*, the additional dataset $D_i$ collected is aggregated to the existing dataset *D* (line 6). The aggregated dataset *D* is used to retrain the policy $\pi _{net}$ with (1) (line 7). After training, a policy $\pi _{net, i+1}$ that causes fewer unsafe or near-collision situations than $\pi _{net, i}$ can be obtained (line 9).

As more data from these problem situations are aggregated, $\pi _{net, i}$ becomes more capable of dealing with the situations^20^. *DAgger* repeats this process until the problem situations rarely happens (line 3). This can be judged by $\hat{\eta }_i$ (line 15 of Algorithm 2) which is the ratio of executed network actions among the total executed actions. If $\hat{\eta }_i$ is greater than the threshold $\eta$, *DAgger* is terminated. Finally, a policy $\pi _{net, i}$ that does not cause unsafe or near-collision situations is obtained (line 8).

#### Data-sampling function in *DAgger*

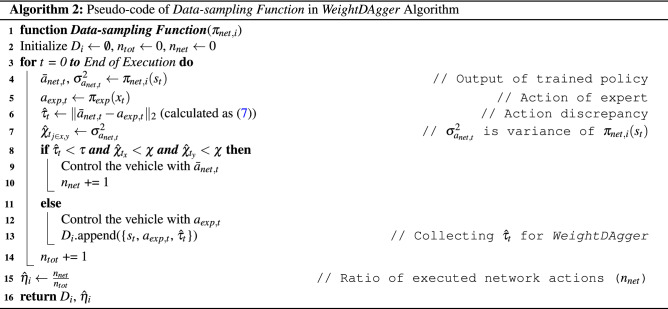



The *data-sampling function* is based on *EnsembleDAgger*^25^. This function quantifies the similarity and confidence for the output of the trained policy $\pi _{net, i}$ to determine whether the driving situation of $\pi _{net, i}$ is unsafe or near-collision. The outputs of $\pi _{net, i}$ and the expert behavior $\pi _{exp}$ are obtained simultaneously (lines 4 and 5 in Algorithm 2) and compared before either is used to control the vehicle (lines 7-9). The discrepancy (error) between the actions of $\pi _{net, i}$ and $\pi _{exp}$ is calculated (line 6), which is defined by the *SafeDAgger* algorithm^26^. To quantify the confidence of $\pi _{net, i}$, the variance of $\pi _{net, i}$ is obtained: $\hat{\chi }_t$ (line 7) used in the *EnsembleDAgger* algorithm^25^.

By determining whether $\hat{\tau }_t$ or $\hat{\chi }_t$ is less than threshold values $\tau$ or $\chi$, an unsafe or near-collision situation can be identified (line 8, see Fig. 4a). In all three situations (see Fig. 4b), $\hat{\tau }_t$ is greater than $\tau$ (blue circle). In the rightmost case, $\hat{\chi }_t$ (red lines) is greater than $\chi$ (blue lines). In these cases, if the vehicle follows the action of the network (red circle), the distance between the vehicle and obstacle decreases, and the possibility of collision increases. In these cases, the expert action (yellow circle) is used to control the vehicle to avoid unsafe situations (line 12). Moreover, only the state $s_t$ of this situation and the expert action $a_{exp, t}$ are collected with the additional dataset $D_i$ (line 13). This is used to intensively train the network to overcome unsafe and near-collision situations.

### *DAgger* with weighted loss function (*WeightDAgger*)

The existing *DAgger* variant algorithms train the policy repeatedly by sampling low-accuracy/confidence data. Based on this algorithm, the proposed *DAgger* training algorithm (*WeightDAgger*) calculates different weights using data as the action discrepancy (through ***Step 1***). These weights are paired with the entire training dataset by comparing the similarity (through ***Step 2***), and the policy is trained with a high learning rate on low-accuracy data. Thus, *WeightDAgger* accurately imitates expert action on these data than existing variants *DAgger* in the same *DAgger* iteration. Consequently, *DAgger* iteration and human effort to collect additional data are reduced.

#### Step 1: Weighted loss function

The weighted loss function is used to address the data imbalance problem in machine learning. In classification tasks, the accuracy of distinguishing classes with a relatively small data proportion is lower than that of classes with considerably more data. The weight is calculated high for the small data class to train the policy with a high learning rate. Therefore, the accuracy for the small data class is similar to that of the large data class.

A weight is defined based on the state in which the weighted loss function is to be applied to imitation learning. During *DAgger* execution, the state accuracy is quantified by calculating the discrepancy between the policy and expert actions. A state with a large discrepancy has out-of-distribution/unseen states in a small proportion in the training dataset. The weight is defined as proportional to the discrepancy, and the policy is trained with a relatively high learning rate for the low-distributed states in the training dataset. Therefore, after training, the accuracy of the state is similar to that of a sufficiently distributed state.

In *WeightDAgger*, the non-weighted loss function $\mathscr {L}_{t_j}$ (2) is replaced by the weighted loss function as follows:

5 $$\begin{aligned} \mathscr {L}_{{{\varvec{W}}}_{t}} = W_t \mathscr {L}_{t}, \end{aligned}$$

where $W_t$ is the weight value, and $\mathscr {L}_{{{\varvec{W}}}_{t}}$ is the weighted loss function. In the policy training process, the change amount of parameter $\theta$ in the policy $\pi (s_i;\theta )$, is calculated as large as the weight $W_t$. $W_t$ can be expressed as follows:

6 $$\begin{aligned} W_t = (1.0 + \alpha \hat{\tau }_t), \end{aligned}$$

where $\alpha$ is the gain for the action discrepancy $\hat{\tau }_t$ mentioned in line 6 of Algorithm 2. $\hat{\tau }_t$ is the normalized value for the distance between the expert action $a_{exp, t_j}$ and the trained policy action $\bar{a}_{net, t_j}$, which can be obtained during *DAgger* execution as follows:

7 $$\begin{aligned} \hat{\tau }_t = \sqrt{\frac{\sum _j (a_{exp, t_j} - \bar{a}_{net, t_j})^2}{n}} \text {, where } \hat{\tau }_t \in [0, 1]. \end{aligned}$$

The numerator in (7) represents the distance between two actions. The actions of each dimension are scaled from 0 to 1; $a_{exp, t_j}$, $\bar{a}_{net, t_j}$ $\in$ [0, 1]. The denominator is used to normalize $\hat{\tau }_t$ from 0 to 1 according to the action’s dimension *n* (e.g., *n*: 2).

The degree to which the policy cannot accurately imitate the expert action in a particular state can be quantified as the action discrepancy $\hat{\tau }_t$. By reflecting $\hat{\tau }_t$ to the loss function $\mathscr {L}_{Gau_t}$ in (2), it is evident that the loss value is larger than that without using $\hat{\tau }_t$ for low-accuracy states, and the policy is trained with a large learning rate. Therefore, a policy trained with $\hat{\tau }_t$ can calculate actions that are more consistent with expert actions than polices trained without $\hat{\tau }_t$.

$\alpha$ in the weight, (6), regulates the application rate of $\hat{\tau }_t$ in the weighted loss function. The higher the $\alpha$, the larger the loss value calculated during training for states where the expert action is inaccurately imitated (large $\hat{\tau }_t$). However, if $\alpha$ is considerably high, the policy may not converge with the lowest loss value, which is similar to failure to converge when using the too large learning rate value of the optimizer. Therefore, the accuracy was experimentally compared by training the policy with different values to determine a proper value for $\alpha$. The results are presented in Section 10).

To apply $\hat{\tau }_t$ to the weighted loss function, $\hat{\tau }_t$ is additionally paired with the additional dataset $D_i$ obtained from the existing *DAgger* algorithms. This pairing process is implemented when obtaining $D_i$ (line 13 in Algorithm 2). In the BC dataset $D_{BC}$, $\hat{\tau }_{t}$ cannot be obtained; thus, all $\hat{\tau }_{t}$ are initialized to zero.

#### Step 2: Weight update process

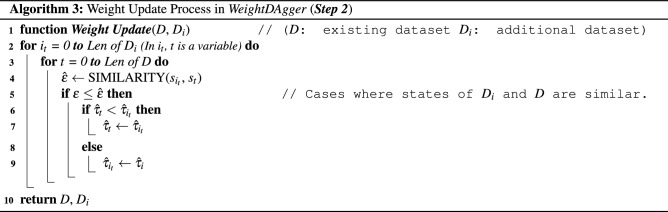



**Figure 5:**
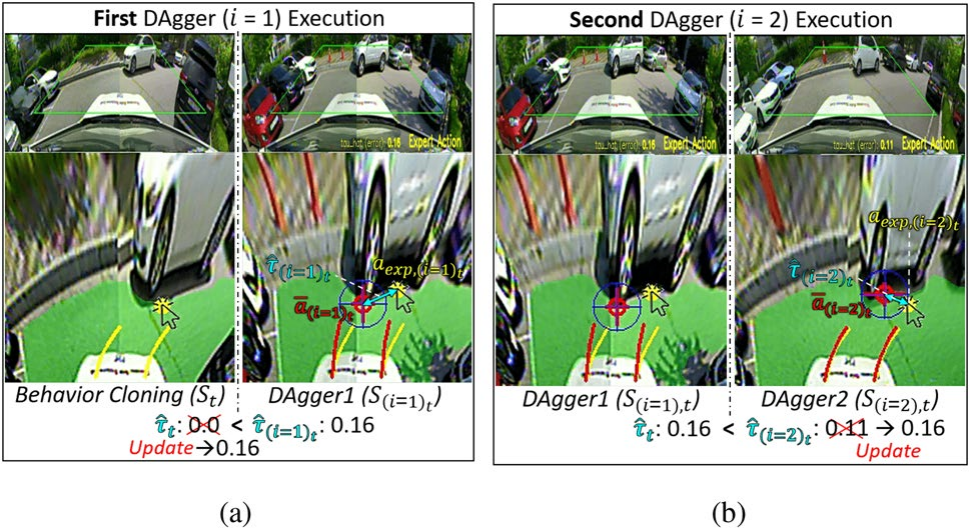
Weight update process (***step 2***) of *WeightDAgger*: (**a**) *DAgger* executed once (*i* = 1) (**b**) Action discrepancy $\hat{\tau }_{{(i=2)}_t}$ still occurs second *DAgger* iterations (*i* = 2).

A weight update process applies to the dataset *D* and the additional dataset $D_i$ collected using *WeightDAgger*. The action discrepancy $\hat{\tau }_t$ in *D* (which is zero) must be updated to a non-zero value to apply the weighted loss function (5) to *D*. Therefore, data exhibiting a high similarity to the states of $D_i$ are searched among the state of *D*, and $\hat{\tau }_t$ among these two data is updated to a larger $\hat{\tau }_t$. This is conducted in Algorithm 3. By using this step, the policy is trained with a weight on all similar data relevant to the situation where the policy cannot accurately imitate expert action.

Algorithm 3 is added to the *DAgger* algorithm (line 5 in Algorithm 1). *D* is the dataset used for training the policy $\pi _{i}$ (Algorithm 2); *t* is the data index in *D*. $D_i$ is the additional dataset obtained through the data-sampling function; $i_t$ is the index of the data in $D_i$. The similarity between $s_{i_t}$ and $s_t$ is calculated and denoted as $\hat{\varepsilon }$ (line 4 in Algorithm 3). Weight updating is conducted when $\hat{\varepsilon }$ is larger than the similarity threshold $\varepsilon$ (line 5). This process comprises two cases (first case lines 6-7 and second case lines 8-9).

In the first case, if $\hat{\tau }_{i_t}$ is greater than $\hat{\tau }_t$ (line 6), $\hat{\tau }_t$ is replaced by $\hat{\tau }_{i_t}$ (line 7), where $\hat{\tau }_{i_t}$ and $\hat{\tau }_t$ are the action discrepancies paired to $s_{i_t}$ and $s_t$, respectively (see Fig. 5a). A large action discrepancy (larger than $\tau$) occurred in the state $s_{(i=1)_t}$, because no sufficient data were similar to $s_{(i=1)_t}$ in the BC dataset. The action discrepancy was paired to $s_t$, which is similar to $s_{(i=1)_t}$ in the BC dataset, via ***Step 2***. Therefore, even with the BC dataset, the policy was trained using the weighted loss function.

In the second case, if $\hat{\tau }_t$ is larger than $\hat{\tau }_{i_t}$ (line 8), $\hat{\tau }_{i_t}$ is replaced by $\hat{\tau }_t$ (line 9). For example, the action discrepancy $\hat{\tau }_{(i=2)_t}$ for $s_{(i=2)_t}$ was slightly reduced after the first *DAgger* iteration (*i* = 1) (see Fig. 5b). Nevertheless, in this situation, the action discrepancy $\hat{\tau }_{(i=2)_t}$ still existed. In ***Step 2***, the policy $\pi _{(t=3)}$ was trained with the weight $\hat{\tau }_t$, which was greater than $\hat{\tau }_{(i=2)_t}$, so that the associated data were trained with a larger weight.

### Reasons to use look-ahead point as action

If the steering-accel/brake is used as the action, the expert suffers two problems in executing *DAgger*, and these can be addressed using the look-ahead point. First, the network action and expert behavior should be obtained simultaneously as shown in lines 4 and 5 of Algorithm 2. When the vehicle is being controlled by a network action, the expert action cannot be obtained simultaneously if the steering accel/brake is used as the action. In the HG-DAgger^27^ data collection process, the joystick (steering wheel and accelerator/brake pedal) must be additionally mounted on the autonomous vehicle. On the other hand, because the proposed method uses the look-ahead point as the action, the expert can select the look-ahead point with only a mouse pointer on the combined image $x_t$ regardless of the network action.

**Figure 6:**
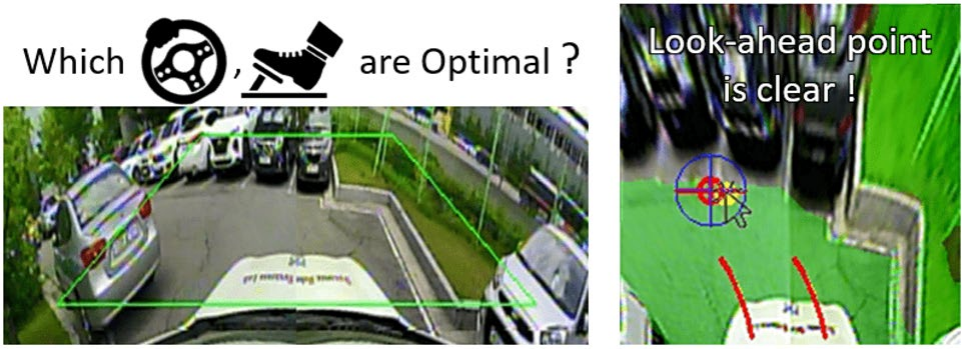
Labeling look-ahead point on the state is more clear than using steer-acc/brake.

Second, the expert cannot clearly and instantaneously find a steering-accel/brake value that the vehicle can drive as safe and fast as possible when performing *DAgger*, even if the action is set as the steering-accel/brake and the expert action can be obtained simultaneously with the network action (see Fig. 6). This is because, when the vehicle is controlled by the network and expert intervention is required, the expert cannot calculate an action value considering the current network action used for vehicle control. Normally when humans drive, they do not directly calculate an absolute steering-accel/brake value, but calculate how much more or less rotate the steering angle and press the accel/brake pedals from the current value (i.e., amount of change).

In this study, the expert selects the look-ahead point that the vehicle should reach on the combined image $x_t$ by referring to the three criteria mentioned in the previous subsection. These criteria specify where the look-ahead point is chosen for $x_t$ by its geometric relationship. Thus, the expert can clearly find one look-ahead point that the vehicle can drive as safe and fast as possible without the current steering-accel/brake feedback of the vehicle controlled by the network. This enables a state action pattern relationship to be clearly identified, so a neural network can learn the driving pattern more clearly.

## Experimental setup

**Figure 7:**
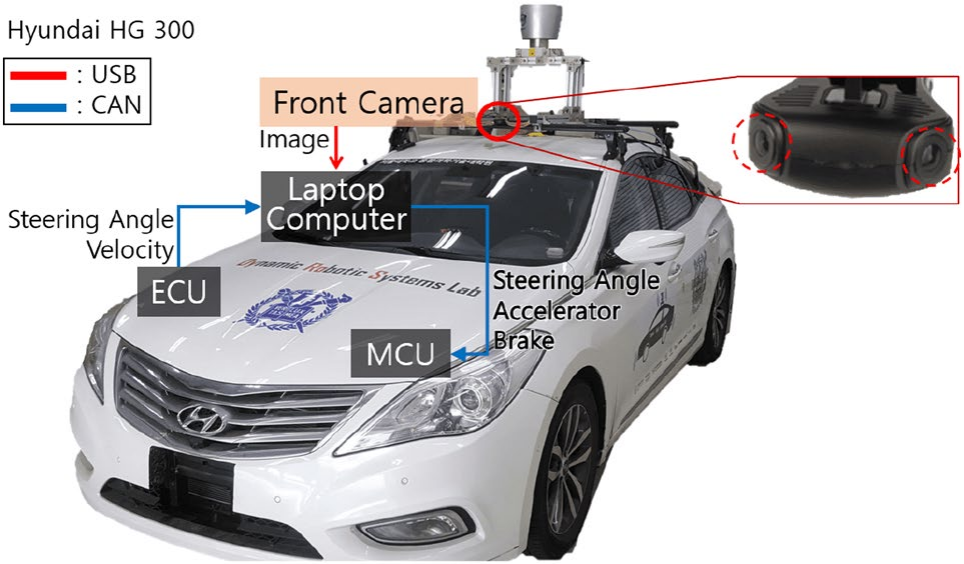
Autonomous vehicle.

The vehicle used in the experiments was a Hyundai HG 240 (Fig. 7). The operating system of the laptop computer was Ubuntu 16.04, and the robot operating system (ROS) was used as a meta-OS platform. The GPU was Nvidia GTX 1080-ti (8 GB), and the CPU was 3.9 GHz Intel i9-8950HK. The steering wheel, accelerator, and brake were controlled by a micro controller unit using a proportional-integral-derivative (PID) controller. A front camera was attached 1.55 m above the ground and 0.25 m forward from the vehicle center. It was rotated about 20$^\circ$ in the pitch direction to minimize the shaded area of the bird’s-eye-view image. This camera comprised two lenses to capture a wide view of the environment. The field of view (FoV) of each lens was 120$^\circ$, and the distortion of images was corrected.

Pure pursuit algorithm^19^ was used to calculate the steering angle command ($\delta$) to reach the look-ahead point: $\delta$ = $\tan ^{-1}\Big (\frac{2L\sin \theta _l}{L_f}\Big )$, where *L* is the wheelbase, and $L_f$ is the distance between the positions of the vehicle and look-ahead point. $\theta _l$ is the look-ahead heading, which is the difference between the heading of the vehicle and the heading of the vector from the vehicle to the look-ahead point. The range of $\delta$ was -540$^\circ$ to 540$^\circ$. The velocity command *v* (m/s) used to reach the look-ahead point was proportional to $a_y$ which is the longitudinal distance between this point and the vehicle. Thus, *v* = $\frac{a_y}{2.24}$, where the final *v* was set to half of $a_y$ for safety reasons. The range of *v* was 0.5 - 2.2 (desired velocity) m/s. The accelerator and brake commands for controlling the velocity were calculated using the PI controller.

### Network training

#### Perception

Softmax cross-entropy was used as the loss function to train the perception network. The drivable and non-drivable probability values were inferred for each pixel, and the average loss of each pixel was calculated^21^. The Otsu algorithm was used to determine the threshold value for drivable probability^28^, which obtains an optimal threshold value at which a probability gap between binary-classified pixels can be smallest. Weights were assigned before training to initialize the network for efficient training. The encoder was initialized with weights trained on ImageNet data. The Adam optimizer with a learning rate of 10$^{-5}$ was used to train the network. The learning rates, 10$^{-6}$, 10$^{-5}$, and 10$^{-4}$ were tested. The training was diverged when the learning rate of 10$^{-4}$ was used, and was converged slowly with 10$^{-6}$. Among 1 $\times$ 10$^{-4}$, 5 $\times$ 10$^{-4}$, 1 $\times$ 10$^{-3}$, the training was converged while preventing overfitting by using the weight decay of 5 $\times$ 10$^{-4}$. In the epoch process up to 100 k, the loss value was converged sufficiently from 10 k. The batch size was set to 128 considering approximately 1000 data. Since we observed adequate empirical convergence with these hyperparameters, we used these values.

**Figure 8:**
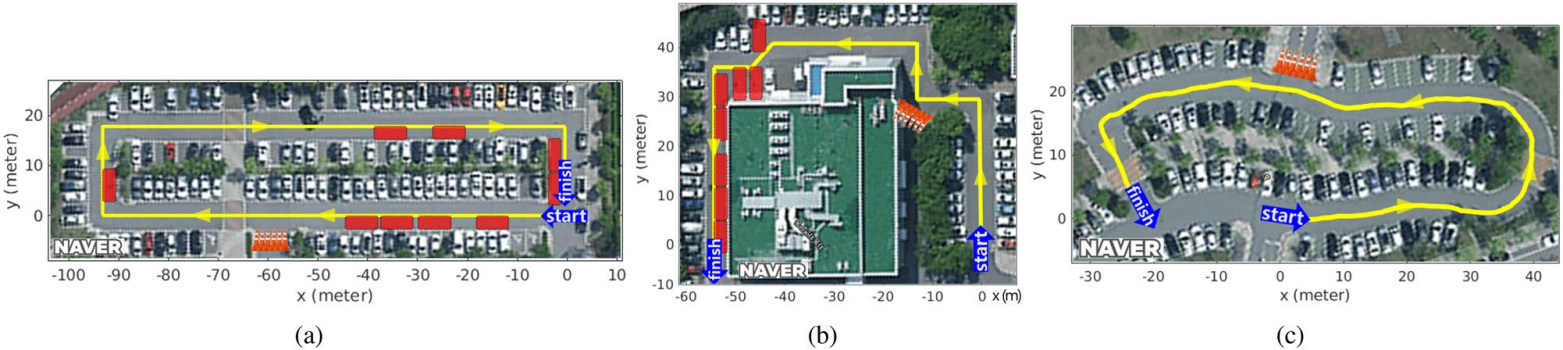
Parking lots used in the real autonomous driving experiment. At intersections, traffic cones are used to guide vehicles to drive in one direction. The yellow line is the center of the drivable area. The red boxes represent obstacle vehicles that were present in the fifth experiment. (**a**) Yellow line is about 230 m long; this parking lot was used to collect the training dataset for imitation learning. (**b**) Yellow line is 139 m long. (**c**) Yellow line is 149 m long.

The training dataset (i.e., RGB-segmented images) was collected for three parking lots as shown in Fig. 8. One image per second was collected for 989 RGB images as the vehicle was driven, and these images were segmented into a drivable and non-drivable class. The RGB and segmented images were transformed into the bird’s-eye-view image and used to train the perception network. Eighty percent of the dataset was used for training, and the rest was used for validation.

#### Driving policy

The deep neural network was used as the driving policy $\pi _{net}$, and it comprised two pairs of convolutional and max-pooling layers with 32 and 64 channels, respectively. The flattened and fully connected layers with 1000 nodes were connected to these layers with 25 % and 50 % dropouts. Finally, the fully connected layer with four nodes was linked to predict the position of the look-ahead point and its confidence. , which is the maximum allowed by the memory of single GPU. The structural-similarity index measure algorithm [29] was used to quantify the similarity between the occupancy grid maps (line 4 in Algorithm 3; see Fig. 15a), and $\varepsilon$ was set 70 $\%$ , which showed the highest accuracy (1.0 - $\hat{\tau }$). The policy was trained with different similarity thresholds $\varepsilon$, and the result is shown in Table 1.

**Table 1:** Accuracy Comparison According to State Similarity Threshold.

		Test Dataset		
		*Accurately Trained State*, $\hat{\tau }_{t}<\tau$	*Inaccurately Trained State*, $\hat{\tau }_{t}\ge \tau$	*Entire State*
State	30	0.9766 {5}	0.9614 {4}	0.9750 {5}
Similarity	50	0.9831 {4}	0.9775 {2}	0.9823 {4}
Threshold	**70**	0.9869 {3}	**0.9844 {1}**	**0.9866 {1}**
$\varepsilon$ (%)	90	0.9872 {2}	0.9631 {3}	0.9847 {2}
	100	**0.9893 {1}**	0.9413 {5}	0.9845 {3}

*Note. The value is the average accuracy. The value in {} is the ranking of the accuracy value in each column.

Significant values are in bold.

In actual autonomous vehicle experiments, *WeightDAgger* based on *EnsembleDAgger*^25^ was used. $\alpha$ parameter in (6) was set to 10. The training dataset for the real autonomous vehicle was collected for the one parking lot shown in Fig. 8a. The vehicle was driven from the start point to the finish point and from the finish point to the start point (totaling 460 m). Data were collected at intervals of 0.05 s as the vehicle was being driven, which was recorded as a video: https://youtu.be/KOXFTEYL-xs. $\tau$ and $\chi$ were set to 0.05 and 0.15, respectively. The final policy was obtained after three *DAgger* iterations (*i* = 3). Increasing the number of *DAgger* iterations can improve performance, but not significantly. The number of collected data were *BC*: 7,082, First DAgger (*i*=1): 12,395, *i*=2: 16,324, *i*=3: 18,704. The ratio of executed network actions ($\hat{\eta }_i$ in Algorithm 2) *i*=1: 0.44, *i*=2: 0.79, *i*=3: 0.91.

### Model-based motion-planning algorithm setup for comparison

The *tentacle*^13^ and *VVF*^16^ algorithms were used to compare with the proposed method, which are matched with a goal of our study to compute in real-time and drive without using global information, and also representative algorithms for the candidate path selection and artificial field methods. The occupancy grid map was used as the input for these algorithms. The steering angle was calculated using each algorithm, and the velocity was set to be inversely proportional to the calculated steering angle.

The *Tentacle* algorithm^13^ has 16 candidate path sets depending on the velocity, and each candidate path set has 81 candidate paths. The cost for each candidate path is calculated using the objective function, and the candidate path with the smallest value is selected. In the experiment, a set of candidate paths of 2.2 m/s was used. The application ratio of the clearance, flatness, trajectory, and forwarding terms in the objective function was; 1:0:0:0.3. The flatness term was not used because the occupancy grid map in this study did not have the occupied probability. Moreover, the trajectory term could not be used because of the absence of global information. When the forwarding term was set to greater than 0.3, the oscillation problem was reduced, but the risk of collision was increased for large curvature changes. The clearance term included a detection range parameter to calculate the proportion of obstacles around the candidate path. This range was set to 0.35 m, which is the width of the vehicle (0.2 m) plus the safety distance (0.15 m). When this was increased further, the vehicle could avoid obstacles more safely, but more oscillation occurred in narrow drivable area.

The *VVF* algorithm^16^ has a repulsive field for obstacles and an attractive field for the goal point, like the artificial potential field algorithm. Additionally, to follow the desired velocity and direction, the velocity field is reflected in the APF field. The look-ahead point is searched by descending along the gradient of the field’s direction from the front of the vehicle to drive along the combined field.

In the experiment, the repulsive, attractive, and velocity fields were set to a ratio of 1:0:0.5. The attractive field could not be used because global path and localization data was not used in this study. The direction of the velocity field was set so that the vehicle could drive forward. When the fields were combined, only the repulsive field was applied around obstacles with a range of 2.3 m. If the range was set greater than 2.3 m, the vehicle could avoid obstacles more safely, but more oscillations occurred when it passed through a narrow drivable area.

### Simulation setup for comparing *DAgger* algorithms

**Figure 9:**
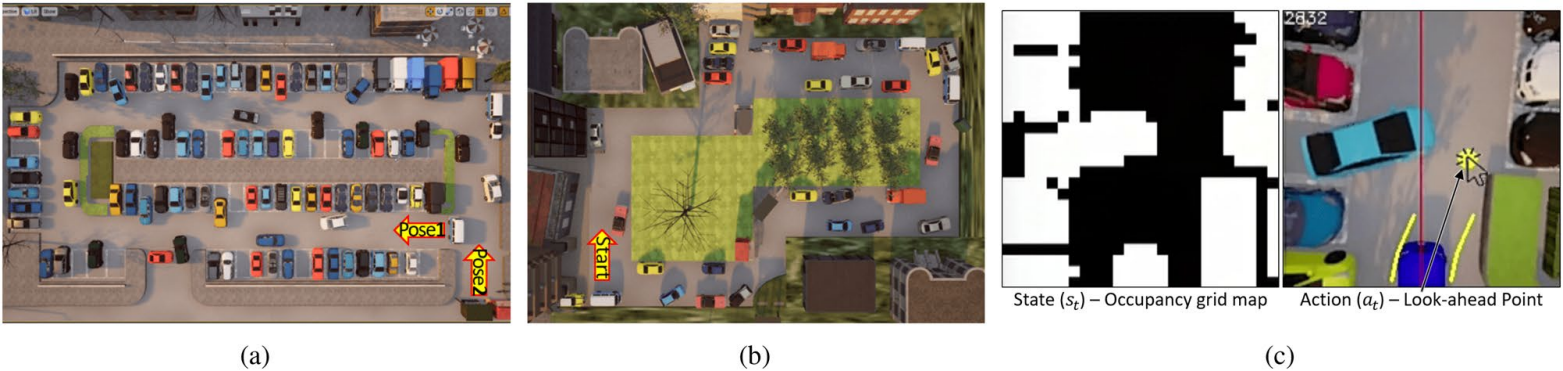
Semi-structured environments created using Unreal Engine4: (**a**) Trained environment (data collection and driving test) (**b**) **Un**trained environment (only driving test) (**c**) State and action in CARLA simulator.

The performance of *DAgger* algorithms was compared in autonomous driving experiments using the CARLA simulator^29^ (see Fig. 9). Parking lots were built using Unreal Engine 4, which had several complex obstacles; the width of the drivable area was narrow, and the change in its curvature was large (see Fig. 9), requiring multiple *DAgger* executions. The occupancy grid map is obtained through a camera installed above the vehicle. We uploaded the environment configurations, code, training dataset, and policies on Github: https://github.com/joonwooahn/WeightDAgger. The existing *DAgger* algorithms were performed in simulation, and *WeightDAgger* was applied to these algorithms. In *VanillaDAgger*^20^, the expert action is used with probability $\lambda ^i\beta _{0} \in [0, 1]$ ($\lambda \in (0, 1)$), so as more *DAgger* is conducted, more policy actions are selected; $\beta _0$ and $\lambda$ were set to 1.0 and 0.5 respectively. *SafeDAgger*^26^ distinguishes whether the trained policy $\pi _{i}$ cannot accurately imitate $a_{exp, t}$ through $\tau$ < $\hat{\tau }_{t}$. *EnsembleDAgger*^25^ also finds the policy action with low confidence: $\tau$ $\le$ $\hat{\tau }_{t}$ or $\chi$ $\le$ $\hat{\chi }_{t_x}$ or $\chi$ $\le$ $\hat{\chi }_{t_y}$. In *HG-DAgger*, the expert took the action when situations were unsafe or near-collision situations^27^. The expert drove the vehicle from Pose1 to Pose2 and Pose2 to Pose1 in only Fig. 9a, and the training data were collected every 0.025 s (see Fig. 9c and video: https://youtu.be/FvuF9gg7_YY)).

## Experimental results

In actual environment experiments, the driving policy obtained through the proposed *DAgger* algorithm (*WeightDAgger*) and model-based motion-planning algorithms were compared. The effectiveness of *WeightDAgger* compared to existing *DAgger* algorithms was verified using CARLA simulation. The reason for using the simulation is to analyze the performance according to the number of collisions and the $\alpha$ parameter in (6). The driving policy was trained separately for each environment.

### Perception network test results

The performance of the perception network was tested with the validation dataset that was not used to train the network. The pixel accuracy was used as the evaluation metric: $\frac{correctly \,\, classified \,\, pixels}{total \,\, number \,\, of \,\, pixels} \, (\%)$ where the numerator is the number of pixels correctly predicted by the network. The result of the pixel accuracy was 98.14 (Fig. 8a), 97.75 (Fig. 8b), and 97.85 Fig. 8c. The drivable area is represented as the green areas in Figs. 11–14. The execution speed of the perception network was 27.9 fps.

### Real environments test: effectiveness of driving policy with proposed imitation learning method

The experiments were conducted in three parking lots without intersections, as shown in Fig. 8. In the driving policy test, the *WeightDAgger* algorithm was analyzed and compared with the *tentacle*^13^ and *VVF*^16^ algorithms: https://youtu.be/OQls9fDgiaA. The calculation from the front camera image through the perception (27.9 ms) and driving policy (20 ms) networks to the look-ahead point value can be completed in about 30 ms.

The *collision rate* was used as an evaluation metric to quantify the performance of each driving policy algorithm. This metric showed the number of collisions per 100 m as the vehicle was driven in each parking lot: $100 \, \frac{cnt_{col}}{len_{path}},$ where $cnt_{col}$ represents the number of times a near-collision situation occurred. When the vehicle headed toward an obstacle and the distance was 0.5 m or less, the vehicle was stopped, and $cnt_{col}$ was incremented. Then, the driving was resumed at a point along the reference path closest to the collision point, as indicated by the yellow line in Fig. 8. At this point, the vehicle could drive without a collision. The length of the reference path was $len_{path}$. A lower *collision rate* indicated a safer driving policy. When the rate was 0, the vehicle could reach the finish point without any collision.

**Table 2:** Collision Rate.

		Fig. 8a Trained Env.	Fig. 8b Untrained	Fig. 8c Untrained Env.
Imitation Learning	***WeightDAgger***	**0 (0)**	**0 (0)**	**0 (0)**
Model-based	*Tentacle*	1.12 (0.95)	1.87 (2.01)	1.47 (1.15)
Motion-Planning	*VVF*	1.38 (1.29)	2.01 (2.15)	1.07 (0.93)

*Note. The average *collision rate* per 100m over 5 trials. The parentheses indicate additional results where the vehicle drove from the finish to start points.

Significant values are in bold.

Table 2 presents the test results for the *collision rate* at the three parking lots over five trials. In the experiment, each algorithm was used to travel a distance of 5180 m. The vehicle using *WeightDAgger* did not encounter any collisions. Even in the untrained parking lot with obstacles of different sizes and shapes, the vehicle drove without any collisions. This result demonstrates that the proposed method has generality. The *tentacle* and *VVF* algorithms resulted in averages of 1.428 and 1.471 collisions per 100 m, respectively. Several unsafe or near-collision (near-collision) situations occurred with the *tentacle* and *VVF* algorithms as described in the next subsections.

**Figure 10:**
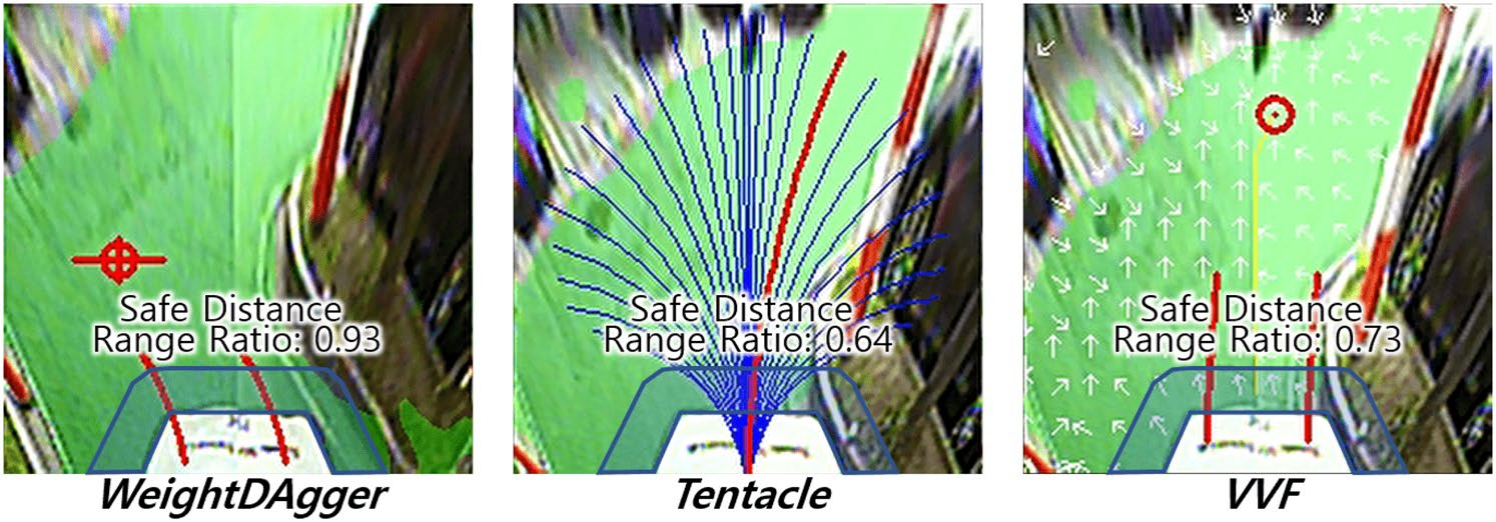
Results of safe distance range ratio; The blue area is the safe distance range, and its ratio is a measure of how much drivable area (green) exist within the blue area.

Moreover, in order to evaluate a collision safety with obstacles, a ratio of the drivable area within 1.0 m range from the end of ego vehicle’s bumper, *safe ratio*, was measured: $\frac{N_{dri}}{N_{ran}},$ where $N_{dri}$ is the number of pixels for the drivable area among $N_{ran}$. $N_{ran}$ is the number of pixels around 1.0 m range from the end of ego vehicle’s bumper, which is indicated in blue range in Fig. 10. By measuring this ratio, we can measure how safely the vehicle can maintain a safe distance from obstacles on average. This range and ratio are shown in Fig. 10 and indicated in Table 3. *WeightDAgger* has the highest safe distance range ratio.

**Table 3:** Safe Distance Range Ratio.

		Fig. 8a Trained Env.	Fig. 8b Untrained Env.	Fig. 8c Untrained Env.
Imitation learning	***WeightDAgger***	**0.83**	**0.72**	**0.91**
Model-based	*Tentacle*	0.69	0.63	0.81
Motion-planning	*VVF*	0.71	0.64	0.85

*Note. The values represent the average *safe distance range ratio* over five trials.

Significant values are in bold.

### Qualitative analysis of driving policies

#### Limitations of *tentacle* algorithm

**Figure 11:**
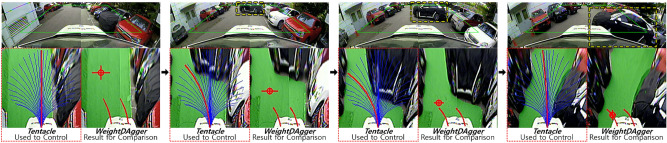
The vehicle using *tentacle* did not drive in the middle of the drivable area and did not avoid obstacles safely. The blue lines in the *tentacle* image represent the candidate paths. The red line represents the selected path to track.

In the *tentacle* algorithm test, the vehicle drove near the boundary between the drivable and non-drivable areas rather than the center of the drivable area after avoiding obstacles or escaping the corner, which is shown in the leftmost image in Fig. 11. This is because the *tentacle* algorithm selects the most forward-facing candidate path with no obstacle among the candidate paths. Then, the vehicle drove at the minimum distance from side obstacles, increasing the possibility of collision. In the same situation, *WeightDAgger* tried to direct the vehicle toward the center of the drivable area. This is because, when the training dataset was collected, experts kept the distance between the vehicle and obstacles as large as possible by considering the overall pattern of the occupancy grid map.

The second to fourth images in Fig. 11 show that, when the vehicle was driving on the side of the drivable area and there was an obstacle in front, the vehicle was unable to avoid the obstacle because of the lack of sufficient space to avoid it. In other situations, even when a vehicle drove along the center of the drivable area and avoided obstacles, it did not avoid the obstacle with sufficient clearance, which is because the *tentacle* algorithm chose the candidate path with the least spacing to avoid obstacles. In contrast, *WeightDAgger* tried avoiding obstacles with sufficient safe distance in advance.

#### Limitations of *VVF* algorithm

**Figure 12:**
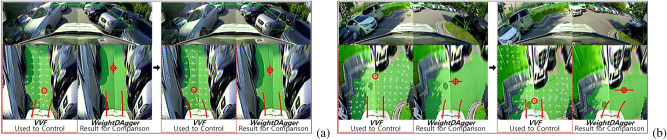
Problems with the *VVF* algorithm; The white arrows represent the field direction. (**a**) Oscillation in a narrow drivable area. (**b**) The vehicle could not enter the right side of the drivable area in advance at a right-angled corner. *WeightDAgger* did not encounter any problems in situations (**a**) and (**b**).

In the *VVF* test, an oscillation problem occurred in narrow drivable areas where the vehicle frequently turned left and right (see Fig. 12a). In such spaces, the magnitudes of the fields from the two obstacles were almost the same because only a repulsive field was applied, but the directions were opposite. Thus, the position of the look-ahead point changed frequently in the opposite directions. This problem may be reduced by decreasing the gain and range of the repulsive force. However, the probability of collision would be increased in other situations, especially where the curvature changed significantly. On the other hand, with *WeightDAgger*, the vehicle drove stably without oscillation by imitating the expert who drove toward the middle of the drivable area even in narrow spaces.

As shown in Fig. 12b, with *VVF*, the vehicle could not enter the drivable area when the curvature changed rapidly, such as right-angled corners. This problem may be addressed using global information, where the goal point would be used as an attractive field. In contrast, this problem did not occur with *WeightDAgger* because when the training set for *WeightDAgger* was collected in this situation, the expert selected a look-ahead point where the vehicle could drive the furthest without collision.

#### Limitations of both *tentacle* and *VVF* algorithms

**Figure 13:**
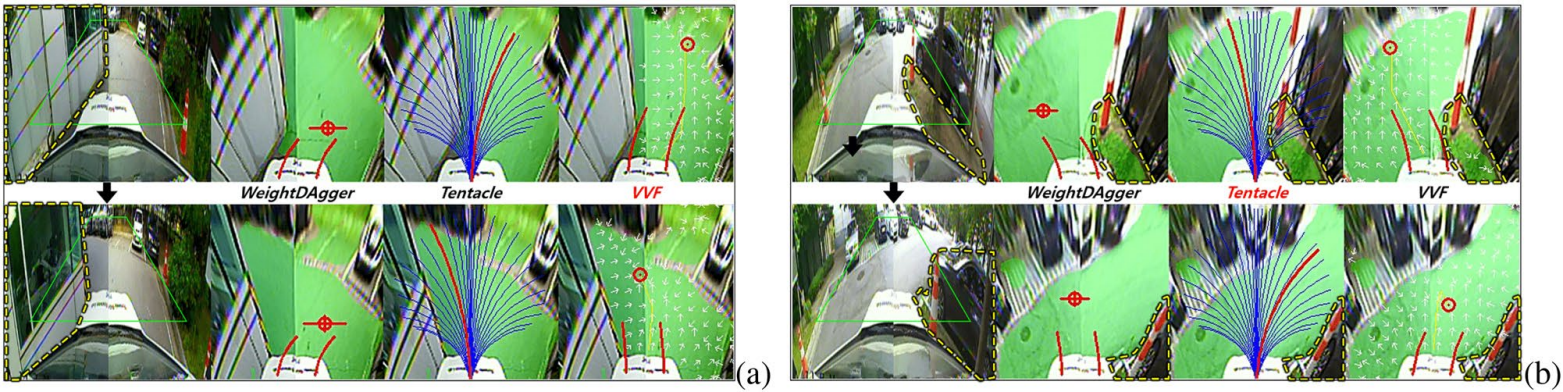
Problems for driving in narrow drivable area with large curvature changes: (**a**) *VVF*, (**b**) *Tentacle*.

Figure 13 shows the problems of the *VVF* and *tentacle* algorithms when the curvature and width of the drivable area changed more than the space where the vehicle was currently driving. The vehicle headed into the drivable area on the side of the adjacent obstacle before sufficiently avoiding it. This is because *tentacle* selected the path with the fewest obstacles among the candidate paths. The candidate path set according to the desired velocity (2.2 m/s) was limited in its ability to handle these situations. For *VVF*, the generated field could not sufficiently consider the nearest obstacles. To address this problem, the range of the repulsive field should be increased. Meanwhile, *WeightDAgger* tried to dodge the nearest obstacle until *WeightDAgger* successfully avoided it because it learned the pattern of preferentially avoiding the nearest obstacles from experts.

#### Driving results on noisy occupancy grid map

**Figure 14:**
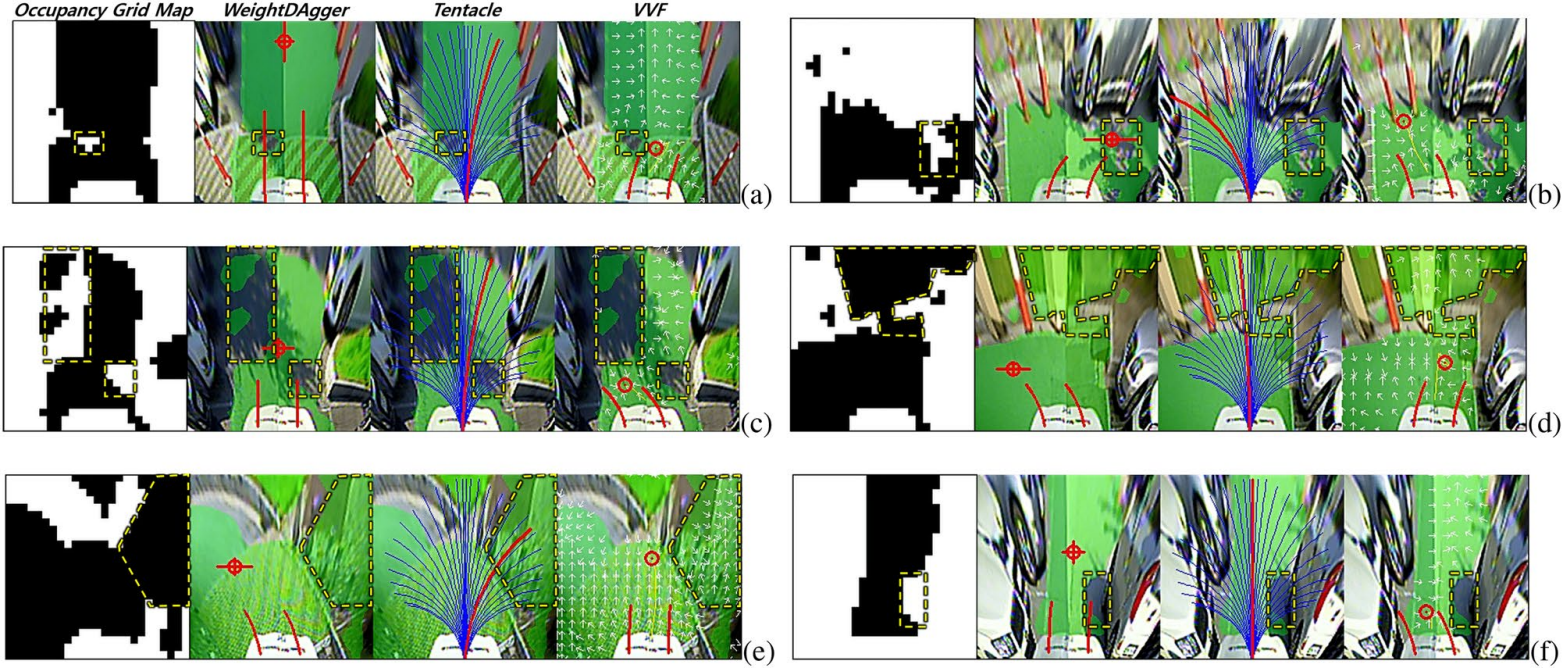
Driving results with *WeightDAgger* when the occupancy grid map contained noise. *WeightDAgger* did not encounter any problems in this situation. However, the vehicle could not drive smoothly or headed towards obstacles with *Tentacle* and *VVF*. (**a**) Noise from misrecognition; (**b, c**, and **g**) Noise by shadow; (**d, e**) Noise at the road boundary.

The occupancy grid map was not recognized accurately in complex and shadowy environments (i.e., noisy input) because the learning data for such situations were insufficient to train the perception network. Data with the noisy state were contained in training data, so the trained network could learn some patterns for the noise and deal with the noisy state. As can be shown from the experiment in Fig. 14, a vehicle could drive without collision, even though there was noise in one trained environment and two untrained environments. However, the *Tentacle* and *VVF* algorithms encountered several problems.

As shown in Fig. 14a, the boundary between the speed bump and road was erroneously recognized as a non-drivable area (i.e., noise). *WeightDAgger* was not affected by this noise because it trained the driving pattern from the overall shape of the state. However, the vehicle drove unstably with the *Tentacle* and *VVF* algorithms to avoid the misrecognized non-drivable area. Figures 14b and c show situations where the noise was caused by shadows. With *WeightDAgger*, the vehicle drove towards the drivable area with fewer oscillations than *tentacle* and *VVF*. This is because a training dataset for *WeightDAgger* contained similar situations, where the expert selected action without being affected by the noise. With the *tentacle* and *VVF* algorithms, however, the vehicle in the Fig. 14b situation avoided the shadows and then drove toward the largest drivable area blocked by obstacles, making it unable to drive any further. These algorithms also had more oscillation problems than *WeightDAgger* especially in Fig. 14c situation.

Figures 14d and e present situations in which a non-drivable area was recognized as a drivable area. In detail, not only the non-drivable area at the curb (i.e., the boundary of the drivable area) but also the space behind the curb was recognized as drivable area. Except for the curb, the vehicle attempted to drive toward the largest drivable area using *WeightDAgger*. However, *tentacle* was influenced by the noise at the curb, which is detected to be a drivable area. So, the vehicle was headed to the curb. *VVF* was less affected than the *tentacle* algorithm, but the vehicle was unable to drive toward the largest drivable area (see Fig. 14d and e). As shown in Fig. 14f, the vehicle with the *VVF* algorithm took actions to avoid the noise caused by a shadow next to the obstacle when passing through a narrow space. For the same situation, *WeightDAgger* and the *tentacle* algorithm did not respond sensitively, and no problem occurred. Estimating a performance of the proposed method in off-road environments, it may be difficult to obtain a robust segmentation result in this environment, but it is expected that the proposed method can be applied because it showed robust driving results against somewhat noisy segmentation results.

### Simulation test: effectiveness of *WeightDAgger*

**Figure 15:**
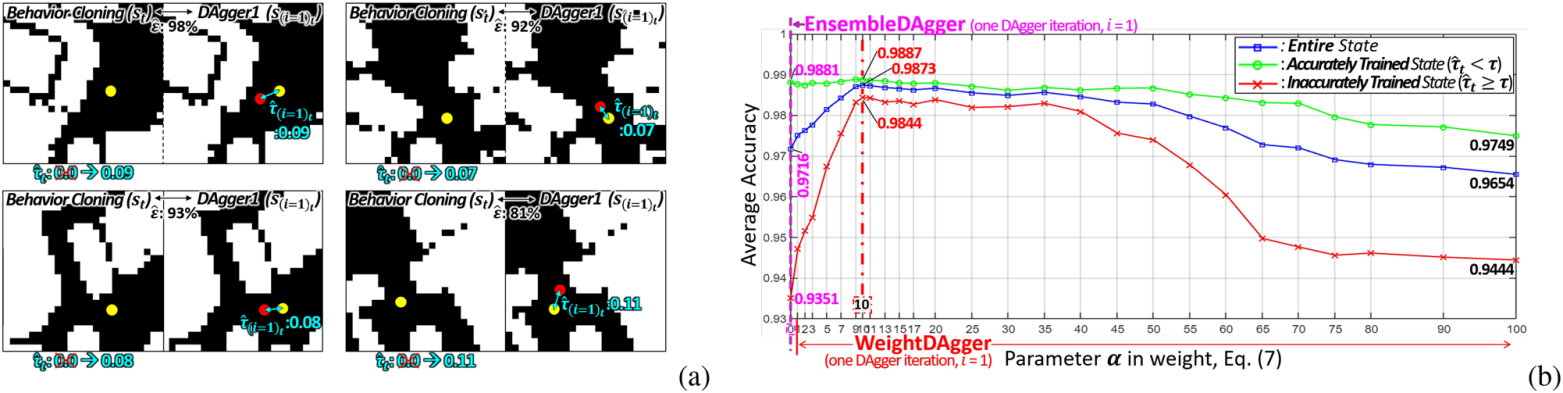
(**a**) Weight update processes (***Step 2***) with $\varepsilon$ = 70% when *DAgger* (e.g. *EnsembleDAgger* in this figure) is executed at once (*i* = 1). (**b**) Results of the average accuracy according to parameter $\alpha$; $\alpha$ of 0 is the same case as *EnsembleDAgger*.

A parameter $\alpha$ in (6) was analyzed. Overall, 80% of the dataset was used as the training set, and the rest was used for the test set. The test dataset was used to measure the accuracy calculated as 1.0 - $\hat{\tau }$ in (7). Additionally, it was classified into two types: accurately and inaccurately trained states. If $\hat{\tau }_{t}$ was less than $\tau$, then this corresponds to the first data type; otherwise, this corresponded to the second data type. The policy was trained by 27 different $\alpha$ (see x-axis of Fig. 15b) with the dataset obtained via *EnsembleDAgger*. In this test, $\varepsilon$ was set to 70. Fig. 15b shows that the accuracies of the accurately trained state (green line) were less sensitive to $\alpha$. For the inaccurately trained state (red line) and entire state (blue line), the accuracy increased with an increase in $\alpha$ from 0 to 10. Conversely, when $\alpha$ was between 10 and 40, the accuracy slightly reduced. If $\alpha$ was excessively large (about 45 or greater), the accuracy was rather low, which is similar to when using the too-large learning rate of the optimizer in the policy training. Overall accuracies were the highest when $\alpha$ was 10.

**Figure 16:**
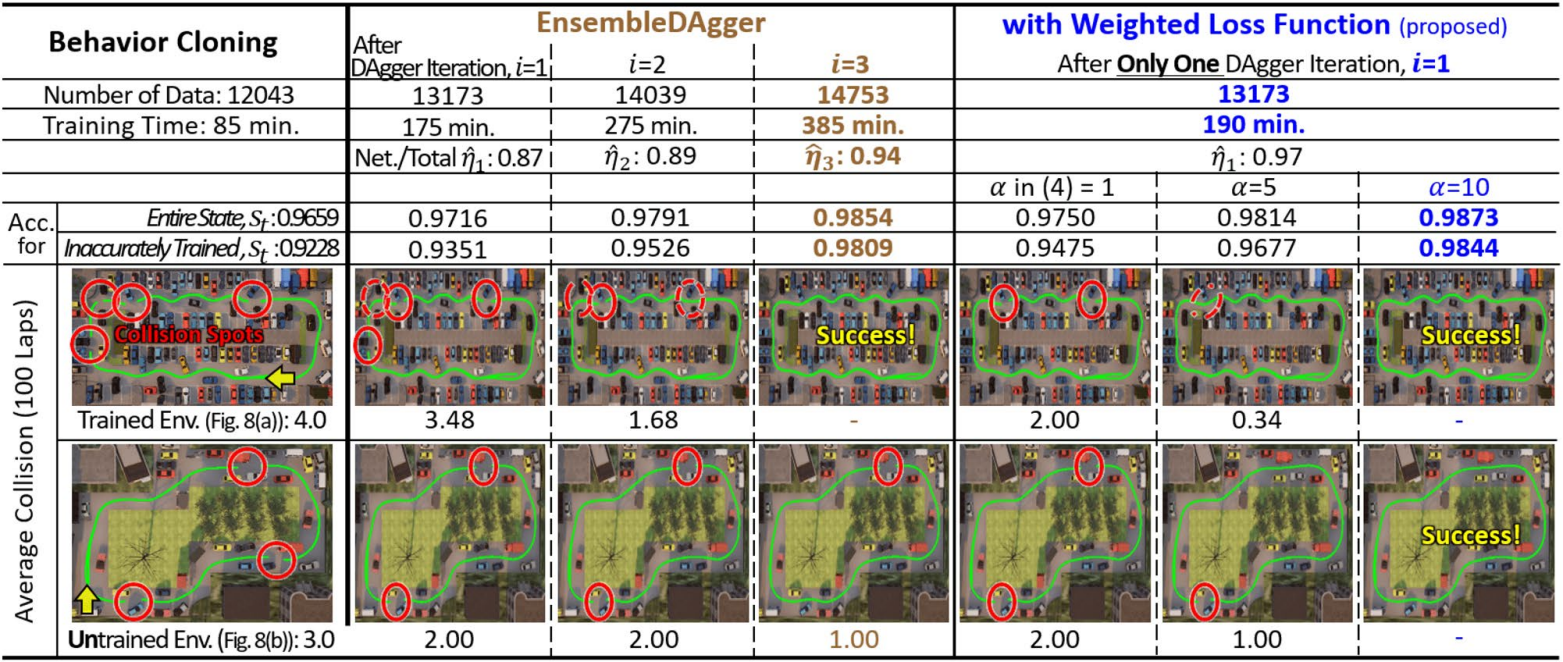
Driving test results; the green line is the vehicle trajectory; the red circle indicates the collision spot; The closer the circle is to the solid line, the higher the collision frequency is. To obtain the policy that the vehicle can be driven without collision, *EnsembleDAgger* with the weight required only one iteration, but original *EnsembleDAgger* needed three iterations.

Figure 16, Table 4, and video: https://youtu.be/O-g4a_xhB3o show the driving results with implemented policies trained using different *DAgger* algorithms. The proposed *DAgger* with the weighted loss function algorithm was used in each of these algorithms, and the average number of collisions encountered within 100 laps was measured. When the vehicle collided with obstacles, driving was resumed 3 m in front of the collision spot on a collision-free trajectory. In both trained and untrained environments, the number of collisions reduced with increased accuracy. The vehicle using a policy trained via *DAgger* variant algorithms with the weighted loss function was driven without collisions in a single *DAgger* execution, except for *VanillaDAgger*. *DAgger* with the weighted loss function trained the policy with a higher weight in collision spots, particularly on roads with narrow or large curvature changes. The existing *DAgger* variants algorithms required more human effort and time to collect additional data than *DAgger* with the weighted loss function.

**Table 4:** Driving Test Results for DAgger variant algorithms and **with Weighted loss function** (proposed)

		*VanillaDAgger*		With **weight**		*SafeDAgger*		With **weight**	*HG-DAgger*		With **weight**	Ensemble -DAgger	With **weight**
After DAgger Iteration, *i*		*i*=1	**i=2**	*i*=1	*i* **=2**	*i*=1	**i=3**	**i=1**	*i*=1	**i=2**	**i=1**	*i* **=3**	**i=1**
Number of Data [ea]		18277	**21076**	18277	**21108**	13094	**14453**	**13094**	13625	**14340**	**13625**	**14753**	**13173**
Training Time [min.]		183	**285**	203	**305**	174	**382**	**188**	177	**280**	**197**	**385**	**190**
Network/Total ($\hat{\eta }_i$)		0.49	**0.76**	0.49	**0.76**	0.85	**0.91**	**0.85**	0.88	**0.94**	**0.86**	**0.94**	**0.69**
Accuracy for	*Entire State*	0.973	**0.975**	0.979	**0.983**	0.970	**0.984**	**0.985**	0.976	**0.983**	**0.986**	**0.985**	**0.987**
	Inacc. Trained	0.931	**0.948**	0.954	**0.978**	0.932	**0.980**	**0.982**	0.945	**0.979**	**0.984**	**0.980**	**0.984**
Average Collision	Trained Env.	3.50	**2.00**	1.50	**–**	3.34	**–**	**–**	2.50	**–**	**–**	**–**	**–**
	Untrained Env.	3.00	**2.00**	2.00	**1.00**	3.00	**1.00**	**–**	2.00	**1.00**	**–**	**1.00**	**–**

*Note. “with weight” means the DAgger with the weighted loss function (proposed). In this test, $\varepsilon$ and $\alpha$ were set to 70% and 10. In “Training Time”, data collection time and training time were included, when GeForce RTX 2080 TI GPU was used to train the policy.

Significant values are in bold.

## Conclusion

In this study, an autonomous driving method using vision-based occupancy grid map and imitation learning is proposed to deal with semi-structured environments such as parking lots. The occupancy grid map obtained via the U-net-based deep neural network was used as an input for imitation learning. Through the geometric relationship between the occupancy grid map and the look-ahead point, the expert clearly labelled this point when collecting the training data, and the *DAgger* algorithm was used for autonomous driving in semi-structured environments. Furthermore, *DAgger* with the weighted loss function (*WeightDAgger*) was proposed to train the driving policy with a high learning rate on high look-ahead point discrepancy data between trained policy and expert.

In real-environment and simulation experiments, a vehicle with the proposed method could drive toward the drivable area while avoiding obstacles reactively in real-time without using a global map and localization. In the actual experiments, the vehicle with *WeightDAgger* could drive more smoothly and safely than with the *tentacle* and *VVF* algorithms in environments where the width and curvature of the drivable area varied significantly. Especially, *WeightDAgger* was more robust when the occupancy grid map was not accurately perceived or was noisy due to a shadow. *WeightDAgger* did not cause any collision, but the *tentacle* and *VVF* algorithms caused 1.42 and 1.47 collisions per 100 m, respectively. This is because the *tentacle* and *VVF* algorithms require different parameters to accommodate different complex situations. In contrast, *WeightDAgger* trains the deep neural network with numerous weight parameters using expert driving data for these situations. Furthermore, simulation test results demonstrates that *WeightDAgger* more accurately imitated expert action, especially for the unsafe or near-collision situations, than without using the weight. Future work will focus on developing proposed method for environments with intersections and dynamic obstacles.

## Data Availability

The dataset used for research and experiments can be obtained through the link below. - Drivable Area Segmentation Dataset: https://url.kr/7pn43m - Imitation Learning Dataset for Actual Vehicle: https://url.kr/h7xv8a - Imitation Learning Dataset for Simulation: https://url.kr/dqm1c3.
